# Novel Cu(II)-based metal–organic framework STAM-1 as a sulfur host for Li–S batteries

**DOI:** 10.1038/s41598-024-59600-8

**Published:** 2024-04-22

**Authors:** V. Niščáková, M. Almáši, D. Capková, T. Kazda, O. Čech, P. Čudek, O. Petruš, D. Volavka, R. Oriňaková, A. S. Fedorková

**Affiliations:** 1 Department of Physical Chemistry, Faculty of Sciences, Pavol Jozef Šafárik University in Košice, Moyzesova 11, 04154 Kosice, Slovak Republic; 2 Department of Inorganic Chemistry, Faculty of Sciences, Pavol Jozef Šafárik University in Košice, Moyzesova 11, 04154 Kosice, Slovak Republic; 3 Department of Chemical Sciences, Bernal Institute, University of Limerick, Limerick, V94 T9PX Ireland; 4 Department of Electrical and Electronic Technology, Faculty of Electrical Engineering and Communication, Brno University of Technology, Technická 10, 616 00 Brno, Czech Republic; 5 Institute of Materials Research, Slovak Academy of Sciences, Watsonova 47, 040 01 Kosice, Slovak Republic; 6 Department of Solid State Physics, Faculty of Science, P. J. Šafárik University, Park Angelinum 9, 041 01 Kosice, Slovak Republic; 7 Centre of Polymer Systems, Tomas Bata University in Zlín, Třída Tomáše Bati 5678, 760 01 Zlín, Czech Republic

**Keywords:** Lithium–sulfur battery, Cathode materials, Metal–organic framework, Energy storage, Energy, Physical chemistry, Materials for energy and catalysis, Batteries

## Abstract

Due to the increasing demand for energy storage devices, the development of high-energy density batteries is very necessary. Lithium–sulfur (Li–S) batteries have gained wide interest due to their particularly high-energy density. However, even this type of battery still needs to be improved. Novel Cu(II)-based metal–organic framework STAM-1 was synthesized and applied as a composite cathode material as a sulfur host in the lithium–sulfur battery with the aim of regulating the redox kinetics of sulfur cathodes. Prepared STAM-1 was characterized by infrared spectroscopy at ambient temperature and after in-situ heating, elemental analysis, X-ray photoelectron spectroscopy and textural properties by nitrogen and carbon dioxide adsorption at − 196 and 0 °C, respectively. Results of the SEM showed that crystals of STAM-1 created a flake-like structure, the surface was uniform and porous enough for electrolyte and sulfur infiltration. Subsequently, STAM-1 was used as a sulfur carrier in the cathode construction of a Li–S battery. The charge/discharge measurements of the novel S/STAM-1/Super P/PVDF cathode demonstrated the initial discharge capacity of 452 mAh g^−1^ at 0.5 C and after 100 cycles of 430 mAh g^−1^, with Coulombic efficiency of 97% during the whole cycling procedure at 0.5 C. It was confirmed that novel Cu-based STAM-1 flakes could accelerate the conversion of sulfur species in the cathode material.

## Introduction

Advances in lithium-ion (Li-ion) battery development technology have increased in recent decades due to the demand for electronic vehicles and the widespread use of Li-ion batteries in electronic devices^1^. Insertion materials used in Li-ion batteries have their disadvantages, and it is necessary to find alternative materials for the cathode with lower cost, higher capacity, and energy density^2,3^. Based on these requirements, lithium-sulfur (Li–S) batteries are offered as an alternative to Li-ion batteries, which have many advantages. Of the many benefits that sulfur offers, the most interesting is its high theoretical capacity (1675 mAh g^−1^). Its theoretical energy density of up to 2600 Wh kg^−1^ (higher than in the case of an NMC type battery (1000 Wh kg^−1^)) is another of its significant advantages. In addition, sulfur is widely available, environmentally friendly, and low-cost compared to the materials used in today's batteries^2,4,5^. Li–S batteries suffer from several disadvantages that prevent their commercialization. One of the biggest problems is that sulfur is an insulator, its conductivity is only around 5 × 10^–30^ S cm^−1^. That is why conductive, mainly carbon matrices, are added to the cathode material to incorporate sulfur, which causes a decrease in energy density. Another problem that must be solved is the solubility of polysulfide intermediates in the electrolyte. The solubility of polysulfides is also associated with the so-called shuttle effect and their migration between electrodes. The formation of polysulfide intermediates further leads to poor mechanical stability, active material loss from the cathode, significant capacity fading, low Coulombic efficiency, and passivation of the lithium surface with Li_2_S/Li_2_S_2_. Mentioned products of the final discharge (Li_2_S_2_/Li_2_S) are placed on the surface of the electrode and create inactive agglomerates/zones, which prevent the transport of ions and electrons in the electrodes. Last but not least, the lithium anode is subject to degradation as a result of surface passivation and the formation of a solid electrolyte interphase (SEI), which causes reduced cycling stability of Li–S batteries^6–9^.

As mentioned above, Li–S batteries face various difficulties that need to be eliminated before commercial use. The cathode, as one of the components of a Li–S battery, is attracting more and more attention from researchers seeking to reduce or remove problems stated before^10,11^. Numerous methods have been studied to improve cathode properties, but the most commonly used way is to incorporate sulfur into the carbon substrate. Several variants of carbon materials have been studied as auspicious sulfur hosts^12^, such as graphene^13,14^, graphene oxide (GO)^15^, reduced graphene oxide (rGO)^16^, and carbon nanotubes^6,17,18^.

Metal–organic frameworks (MOFs) are attracting progressive attention as one of the most promising materials for batteries due to their variable composition, extraordinary surface areas, and properties^19,20^. MOFs are organic–inorganic hybrid materials that form coordination bonds between central metal ions and organic ligands^21–23^. The type of ligand and its properties (bond angles, ligand length, bulkiness, chirality, etc.) play an important role in the resulting MOF structure. Also, the choice of the central atom, especially the preferred coordination number, shape, and ability to form clusters of different shapes, significantly impacts the resulting design of the MOF framework^22,24–26^. MOF structures have uniform and well-ordered pore sizes and very high surface areas compared to many carbonaceous materials used as hosts for sulfur in Li–S batteries. Previous studies have shown that central metal ions containing coordinatively unsaturated sites and organic ligands can efficiently adsorb polysulfides within MOFs pores^27^. Porous coordination polymers have unique crystal structures, large internal surfaces, and tunable cavities^22^. Their most significant advantage is their porosity (up to 90% free volume) and diversity in composition. Due to their high porosity and specific surface, more accessible mass transport and diffusion are possible, and their smaller pores allow the adsorption and separation of small molecules^21,23^. MOF can be used to improve cathode materials and other battery components, such as anode or electrolyte materials^28^. Their structure is easily adjustable by simply controlling synthesis parameters such as starting chemicals, synthesis conditions (temperature, pH value, concentration, etc.), and preparation methods (hydro/solvothermal synthesis, diffusion, mechanochemical and electrochemical synthesis, microwave-assisted preparation, etc.)^29–31^. MOF properties can be improved by combining them with various materials, such as carbon^32^, polymers^33^, graphene^34^, enzymes^35^, and metal nanoparticles^22,36^. Nowadays, a large number of MOFs are known, and their number is still increasing. This fact also points to the ongoing interest of research groups around the world in the application of MOF materials in various fields. Due to their interesting properties and modifiable surface area, MOF materials are widely used not only in the field of energy storage^28,37^ and batteries^38,39^ but also in the field of gas adsorption and separation^40–42^, heterogeneous catalysis^43–45^, drug delivery^46,47^, and many other areas^48–52^.

This work presents the synthesis, characterization, and application of MOF represented by STAM-1 as cathode material in Li–S batteries. The essential structural components of the compound are Cu(II) paddle-wheel cluster and monomethyl ester of benzene-1,3,5-tricarboxylic acid. The material was prepared using the solvothermal method, during which a mono-esterification reaction of one of the three carboxyl groups of the linker takes place during the synthesis and significantly impacts the resulting STAM-1 framework. The uniqueness of the material lies in the bimodal pore size and shape in the STAM-1 skeleton, which contains hexagonal non-polar and trigonal polar pores. Moreover, using copper can enable a Cu(II) cation to offer two Lewis acid sites, improving its properties to hold more sulfur. In the past, our research group published studies dealing with the entrapment of polysulfides in MOF materials, specifically MIL-101(Fe)-NH_2_^53^ and MOF-76(Gd)^38^. The main advantage of these materials was the mesoporosity in the case of MIL-101(Fe)-NH_2_ and the transformable framework of the MOF-76(Gd) compound. The key difference compared to previous studies is the use of STAM-1, as it contains pores with different polarity. As the old, the well-known saying applies: "Like Dissolves Like". This means that polar compounds dissolve polar compounds, and nonpolar compounds dissolve nonpolar compounds. Polar (hydrophilic) pores are suitable for capturing polysulfides with a short chain, and elemental sulfur or polysulfides with a longer non-polar chain will prefer interaction and encapsulation in non-polar (hydrophobic) pores. By inserting STAM-1 into the electrode material, we wanted to prevent the "shuttle effect", which is the main reason for capacity decay. Successful synthesis, activation temperature, and textural properties (BET surface area, pore volume) were investigated by combining CHN elemental analysis, heating infrared spectroscopy (IR), thermogravimetry (TG), powder X-ray diffraction (PXRD), X-ray photoelectron spectroscopy (XPS) and nitrogen and carbon dioxide adsorption measurements. STAM-1 was further applied as a precursor to construct S/STAM-1/Super P/PVDF cathode material for the Li–S battery. The morphological structure of prepared STAM-1 and fabricated S/STAM-1/Super P/PVDF cathode were also checked by scanning electron microscopy (SEM). For the electrochemical characterization of the assembled cathode, cyclic voltammetry, electrochemical impedance spectroscopy, and galvanostatic cycling were used. The outcomes of galvanostatic cycling showed that the initial capacity of this cathode material was 495 mAh g^−1^ at 0.2 C and, after one hundred cycles, 430 mAh g^−1^ at 0.5 C. The electrode we prepared shows a high Coulombic stability of 96% after 100 cycles. Through this experimental fact and the results of electrochemical impedance spectroscopy, our hypothesis regarding the storage of soluble polysulfides in polar and non-polar cavities of STAM-1 to prevent the "shuttle effect" was confirmed.

## Experimental part

### Chemicals

Sulfur (Sigma-Aldrich), carbon Super P (Alfa Aesar), PVDF (polyvinylidene difluoride) (Sigma-Aldrich), copper(II) nitrate trihydrate (Sigma-Aldrich), benzene-1,3,5-tricarboxylic acid (Sigma-Aldrich), methanol (CentralChem), NMP (N-methyl-2-pyrrolidone) (Sigma-Aldrich), metal lithium (Sigma-Aldrich), glass fiber-based separator (Whatman), lithium nitrate (LiNO_3_) (Acros Organics), lithium bis(trifluoromethanesulfonyl) imide (LiTFSI) (Sigma-Aldrich), 1,2-dimethoxyethane (DME) (Sigma-Aldrich), 1,3-dioxolane (DOL) (Sigma-Aldrich), ethanol (Centralchem).

### Synthesis of STAM-1

The STAM-1 porous material was prepared by modifying the synthetic procedure described in^54^. 1 g (4.14 mmol) Cu(NO_3_)_2_·3H_2_O and 0.87 g (4.14 mmol) benzene-1,3,5-tricarboxylic acid (H_3_BTC) in a molar ratio of 1:1 were dissolved in 20 cm^3^ of a mixed solvent H_2_O/MeOH (volume ratio 50:50) in Teflon-lined steel autoclave Parr. After dissolving the reactants, the autoclave was closed and placed in an oven with controlled heating, while the temperature regime was set as follows: The reaction mixture was heated with a heating rate of 0.5 °C min^−1^ to 110 °C for 168 h (7 days) and then cooled with a cooling rate of 1 °C min^−1^. After cooling to ambient temperature, the STAM-1 material in the form of a blue powder was filtered, washed with methanol, and dried in a stream of air. The weight of the prepared product was 2.48 g, representing a yield of 88%. Elemental analysis for STAM-1 (Cu_2_C_20_H_24_O_19_, 679.48 g mol^−1^): exp.: 35.18% C, 3.61% H; clcd.: 35.35% C, 3.56% H. IR with the assignment of characteristic vibrations (in cm^−1^): υ(OH) 3448; υ(CH)_ar_ 3088; υ(CH)_aliph_ 2998, 2954; υ(C=O) 1731, 1713; υ(COO^−^)_as_ 1638; υ(C=C)_ar_ 1589; υ(COO^−^)_s_ 1376; δ(COO^−^) 730.

### Preparation of S/STAM-1/Super P/PVDF and cell assembly

STAM-1 was used as an additive in the sulfur cathode material in Li–S batteries. Firstly STAM-1 was activated in an oven at 150 °C and kept at this temperature for 30 min. Before the activation, STAM-1 had a turquoise color; after the activation process, STAM-1 slightly darkened, which indicated successful dehydration. After the activation process, a composite of sulfur, carbon Super P, and STAM-1 was prepared by milling in a planetary ball mill. Components were filled into a zirconium grinding jar in a mass ratio of 66:17:17 and milled with the addition of ethanol at 150 rpm for 60 min using 10 zirconium balls, each approximately 1 cm in diameter.

After mixing, electrode slurry was prepared by stirring the above-mentioned composite and polyvinylidene fluoride (PVDF) in *N*-methyl-2-pyrrolidone (NMP). The slurry was finally composed of sulfur, carbon Super P, activated STAM-1, and PVDF in the weight ratio of 60:15:15:10. The slurry was stirred for 24 h to homogenize the prepared electrode material thoroughly. After 24 h of stirring, the slurry was coated using the coating bar on an aluminium foil with carbon surface modification. The coated electrode material was dried in an oven at 60 °C for 24 h. The measured sulfur content in the composite material is 58.65%, and the value is close to the theoretically suggested (60%). The difference is probably due to insufficient drying of the slurry at 60 °C for 24 h since the boiling point of the solvent used in the preparation of the electrode material, *N*-methyl-2-pyrrolidone (NMP), is 202 °C. Its presence was manifested by increased carbon/hydrogen content and the presence of nitrogen atoms (expected CHNS values: C 23.55%, H 0.85%, N 0.00%, S 60.00% and measured CHNS values: C 25.68%, H 1.37%, N 0.29%, S 58.65%). The electrodes were then cut out with a diameter of 18 mm and pressed with the hydraulic press with a pressure of 350 kg cm^−2^. The electrodes were dried in an argon atmosphere at 60 °C for 24 h. Loading of sulfur was 2.35 mg cm^−2^. The test cells (El-Cell®) were assembled in the argon-filled glove box (Jacomex) for electrochemical testing. Prepared electrodes were used as working electrodes, and metal lithium was used as a counter and reference electrode. Glass fibre separator was used as a separator, and 0.25 M of lithium nitrate (LiNO_3_) + 0.7 M of lithium bis(trifluoromethanesulfonyl) imide (LiTFSI) solution in 1,2-dimethoxyethane (DME) and 1,3-dioxolane (DOL) with the volume ratio DME:DOL 2: 1 was impregnated into this separator.

### Material characterization

Elemental analysis was performed using a CHNOS Elemental Analyzer Vario MICRO instrument. Infrared spectra of STAM-1 at ambient temperature and after heating were carried out on a Nicolet 6700 instrument in the wavelength range of 4000–400 cm^−1^ using the KBr technique. Thermal analysis was performed on a NETZSCH STA 449 F3 Jupiter analyzer in an atmosphere of argon with a flow rate of 60 cm^3^ min^−1^ in the 20–600 °C temperature range and a heating rate of 10 °C min^−1^. Nitrogen (99.999% purity) and carbon dioxide (99.995% purity) adsorption measurements at − 196 and 0 °C, respectively, were performed on a Micromeritics 2020 Plus instrument. Before gas adsorption, the sample was degassed at a temperature of 150 °C for 12 h under a vacuum. The surface area of STAM-1 was calculated using BET (Brunauer-Emmet-Teller) and DR (Dubinin-Radushkevich) methods from nitrogen and carbon dioxide adsorption data, respectively. For scanning electron microscopy (SEM) and energy-dispersive X-ray spectroscopy (EDX) characterization, a scanning electron microscope Vega3 Tescan with an EDAX analyzer was used. Powder X-ray diffraction (PXRD) was measured using a diffractometer (model Rigaku MiniFlex 600) equipped with a Cu Kα radiation (*λ* = 1.5406 Å) in 2*θ* range of 5–60°. Raman spectra were collected using an XploRA ONE spectrometer (Horiba Yvon Jobin, France) equipped with a 532 nm laser (power 50 mW). The accumulation time was 10 s with double repetition. High-resolution XPS measurements were carried out on a SPECS PHOIBOS 100 analyzer using an Al anode operating at 200 W and a base pressure of 10^–8^ mbar. The samples were placed in a molybdenum sample holder using conductive carbon tape.

### Electrochemical characterization

The electrochemical properties were examined by electrochemical impedance spectroscopy (EIS), cyclic voltammetry (CV), and galvanostatic cycling measurements using Autolab potentiostats (PGSTAT101 and Autolab MAC90166). EIS was measured in the frequency range of 1 MHz–100 mHz with an amplitude of 10 mV. EIS spectra were fitted in MATLAB software using the ‘Zfit’ function^55^. The CV was realized in the potential window from 1.8 to 2.8 V with scan rates of 0.1 mV s^−1^ and 0.2 mV s^−1^. Galvanostatic cycling was performed within the voltage range between 1.8 and 2.8 V versus Li^+^/Li at different C-rates.

## Results and discussion

### Crystal structure of STAM-1

**Figure 1:**
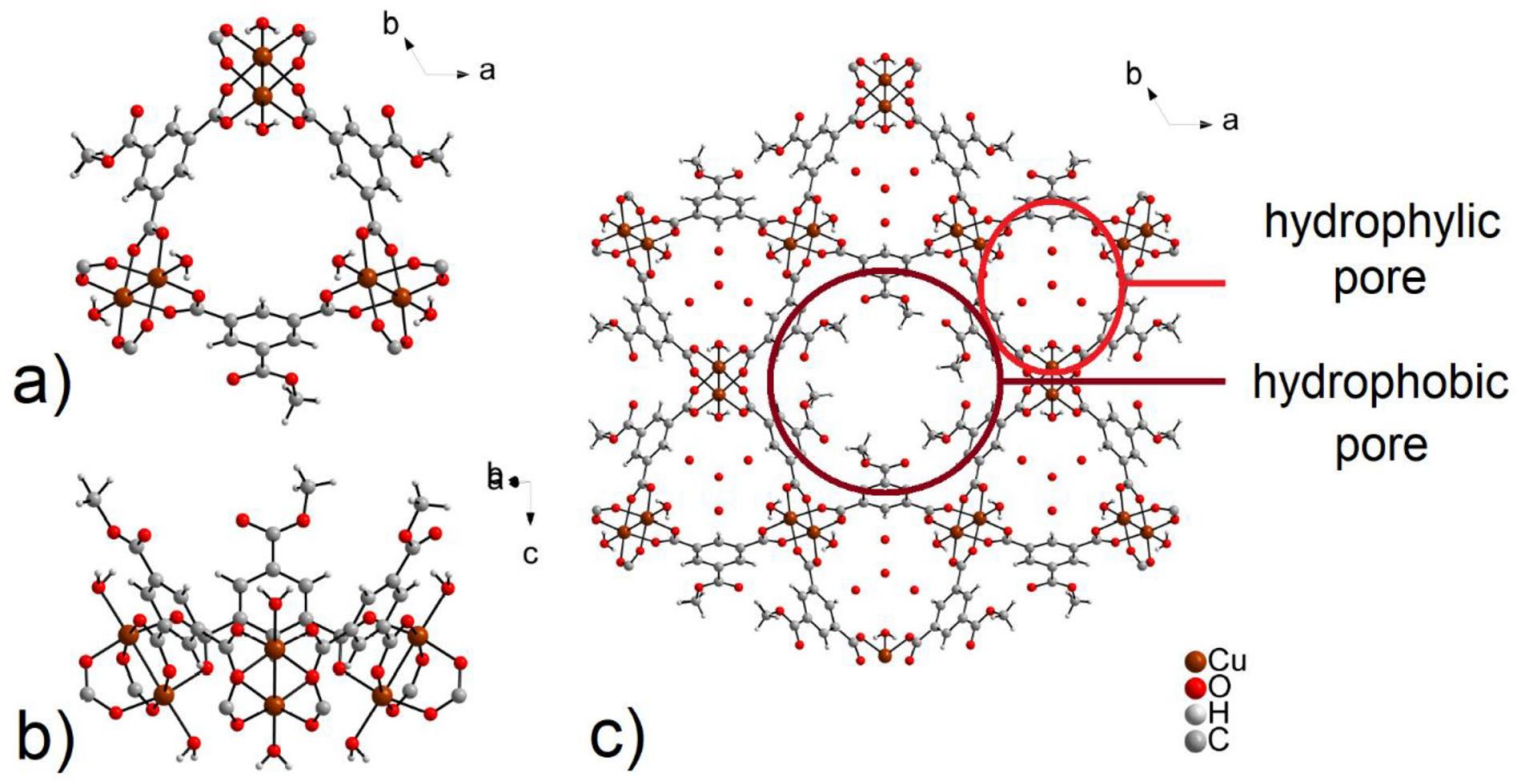
(**a**) Paddle-wheel clusters bridged by MeBTC linkers in STAM-1 (crystallization water molecules are omitted for clarity). (**b**) Mimetic calixarene structure of hexanuclear unit with non-polar part formed by phenyl ring and methyl ester groups (up) and polar part formed by paddle-wheel cluster (down). (**c**) A view of hydrophobic and hydrophilic pores present in the STAM-1 framework.

The asymmetric unit of STAM-1 consists of one monomethyl ester of BTC linker (MeBTC(-II)), one Cu(II) central ion, and one coordinated and two crystallization water molecules. Cu(II) cations in STAM-1 formed a paddle-wheel cluster with a square secondary building unit, similar to HKUST-1. The cluster is formed by two terminally coordinated water molecules and four carboxylate groups coordinated in *syn-syn* mode originating from four MeBTC linkers. One MeBTC molecule forms a bridge between two paddle-wheel clusters (see Fig. 1a). The difference between the well-known HKUST-1 and STAM-1 is that during the synthesis of STAM-1, an esterification reaction of one carboxyl group of benzene-1,3,5-tricarboxylic acid occurs, which results in a different geometry of the resulting framework. The result is a building block that mimics calixarene and contains a polar and non-polar part (see Fig. 1b). The uniqueness of STAM-1 lies in the presence of hydrophobic and hydrophilic pores in the MOF´s framework (see Fig. 1c). Ester functional groups form a hexagonal hydrophobic cavity, the channels propagating along the *c* crystallographic axis and have a size of 4–5.65 Å. Hydrophilic pores have a trigonal shape in which crystallization water molecules are located, and the size of the channels is 4 Å. The presence of channels with a different polarity led us to apply the STAM-1 compound as a sulfur carrier for Li–S batteries.

### STAM-1 characterization

**Figure 2:**
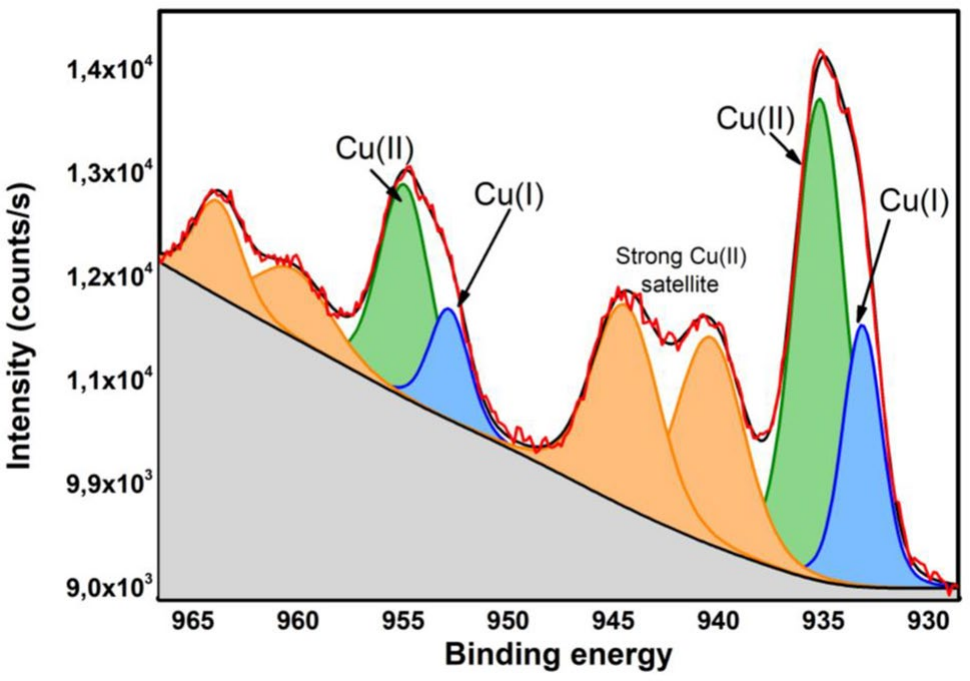
The high-resolution Cu 2p XPS spectrum of STAM-1.

The oxidation state of copper in STAM-1 was determined by X-ray photoelectron spectroscopy (XPS), and the measured Cu 2p XPS spectrum is shown in Fig. 2. The Cu 2p3/2 peak was fitted with two components corresponding to Cu(I) oxidation state with binding energy at 933.16 eV and Cu(II) oxidation state at 935.19 eV. The same set of components fits the Cu 2p1/2 peak. We assigned the component with binding energy at 952.81 eV to Cu(I) 2p1/2 and the component at 954.94 eV to Cu(II) 2p1/2. The spin–orbit splitting for the Cu(I) oxidation state obtained from fit is 19.65 eV and 19.75 eV for Cu(II) oxidation state. The Cu(I)/Cu(II) ratio of 0.32 was determined from the Cu 2p3/2 peak. The strong satellite peaks characteristic of the oxidation state of Cu(II) are also recognizable^56,57^ .

**Figure 3:**
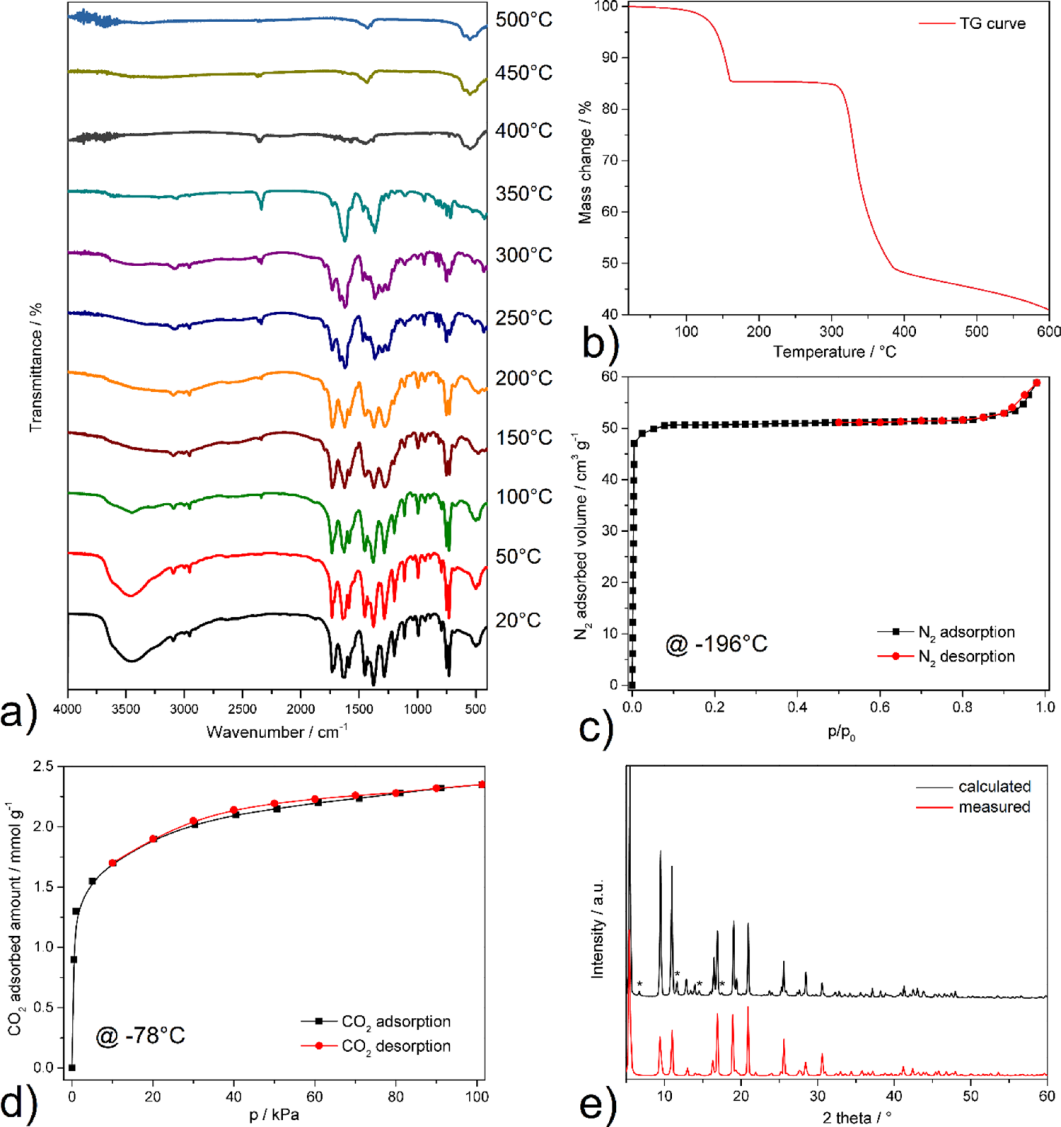
(**a**) Heating IR spectra of STAM-1 measured at 20, 50, 100, 150, 200, 250, 300, 350, 400, 450, 500, 550, and 600 °C. (**b**) Thermogravimetric curve measured in an argon atmosphere in the temperature range of 20–600 °C. (**c**) Nitrogen adsorption/desorption isotherm measured at − 196 °C. (**d**) Carbon dioxide adsorption/desorption isotherm measured at − 78 °C. (**e**) Comparison of the measured and calculated PXRD pattern from the STAM-1 crystal structure (* the diffraction peaks belonging to HKUST-1 as impurity).

The presence of building blocks and guest molecules in STAM-1 was confirmed by IR spectroscopy (see Fig. 3a, black line). In the infrared spectrum of the STAM-1 compound, a broad absorption band at 3448 cm^−1^ can be observed, which corresponds to the stretching vibration (υ(OH)) of coordinated and crystallization water molecules. The presence of the MeBTC linker is evident by the presence of many characteristic absorption bands. The methyl ester was manifested in the IR spectrum by the aliphatic stretching vibrations of the CH methyl group at 2998 and 2954 cm^−1^ and the absorption bands of the carbonyl group (υ(C = O)) at 1731 and 1713 cm^−1^. The phenyl ring of MeBTC was displayed by the aromatic absorption band (υ(CH)) at 3088 cm^−1^ and the stretching vibration of C = C at 1589 cm^−1^. The carboxylate group in the paddle-wheel cluster is present by the absorption bands of symmetric and asymmetric stretching vibrations of carboxylate groups at 1638 cm^−1^ for υ(COO^−^)_as_, 1376 cm^−1^ for υ(COO^−^)_s_ and deformation vibration δ(COO^−^) at 730 cm^−1^.

The thermal stability and dehydration process of STAM-1 were studied by a combination of thermal analysis and heating infrared spectroscopy. Thermal analysis of STAM-1 was carried out in the temperature range of 20–600 °C in an argon atmosphere. As is evident from Fig. 3b, in the temperature interval 20–150 °C, three water molecules were released, which showed a 15.3 wt% mass loss on the TG curve (clcd. mass change 15.9 wt%). The dehydrated compound is subsequently stable up to 300 °C due to a plateau on the TG curve. Further heating causes thermal decomposition of the STAM-1 polymeric framework. Since the analysis was carried out in an argon atmosphere, complete decomposition of the organic part of STAM-1 did not occur at 600 °C, and the presence of carbonized products was reflected by a higher residual mass on the TG curve (obs. 41.2 wt%, clcd. 23.4 wt% for CuO as final decomposition product). Figure 3a shows the IR spectra of the compound measured by gradual heating of a KBr pellet in the temperature range of 20–600 °C with 50 °C increments. The sample, in the form of a KBr tablet, was heated in an oven, kept at the given temperature for 10 min, and then the IR spectrum was measured. As is evident, the dehydration of STAM-1 occurs at a temperature of 150 °C. The mentioned process was manifested by a gradual decrease in υ(OH) intensity at 3448 cm^−1^. Subsequently, the compound is stable up to 300 °C, as no significant changes in intensity or absence of characteristic absorption bands are observed in the IR spectra. Further heating at a temperature of 350 °C leads to the decomposition of the methyl ester groups, as a decrease in the intensity of methyl groups (υ(CH)_aliph_) at 2998 and 2954 cm^−1^ and the carbonyl functional group (υ(C = O)) at about 1700 can be observed in the IR spectrum. In the temperature range of 400–500 °C, the carbonization of the MOF´s organic skeleton occurs, and novel absorption bands at 592 and 539 cm^−1^ are present, which belong to copper(II) oxide as the final decomposition product^58^. From the described results of TG and IR spectroscopy with gradual heating, the results of both methods are complementary and in good agreement. The described measurements proved that the dehydration of STAM-1 and the opening of the framework occurs after heating to 150 °C.

The porosity of STAM-1 was studied by the adsorption measurements of nitrogen at -196 °C (see Fig. 3c) and carbon dioxide at − 78 °C (see Fig. 3d). Before the measurements, the sample was activated at 150 °C under a dynamic vacuum for 12 h, while nitrogen adsorption was initially measured and CO_2_ adsorption was performed on the same batch of the sample as N_2_ adsorption. Due to the structural diversity of pores in the framework and different surface chemistry caused by the presence of hydrophobic and hydrophilic pores, the STAM-1 adsorption measurements show different textural properties than those determined from molecular simulations. Theoretical studies have shown that the available surface area in STAM-1 is 420 m^2^ g^−1^ (pore volume 0.24 cm^3^ g^−1^), while the model calculation was carried out by rolling nitrogen molecules over the surface of STAM-1 determined from the crystal structure analysis^54^. The nitrogen adsorption/desorption isotherm of STAM-1 shows almost ideal type *I* behavior according to the IUPAC classification^59^, which is characteristic of microporous materials. In the case of microporous materials, the pores are filled with adsorbate molecules at low relative pressures, which results in a sharp increase in the adsorbed volume. Subsequently, a plateau is observed on the adsorption isotherm at higher pressure values, as all available pores are filled with nitrogen molecules. The desorption isotherm often describes the shape of the adsorption branch, as was the case for STAM-1. From the measured adsorption data in the interval *p/p*_*0*_ = 0.05–0.2, the BET area was calculated with a value of 207 m^2^ g^−1^. By comparing the measured and calculated surface area, it follows that the value is almost twice lower than the calculated one, indicating that only one type of pores is accessible for the selected adsorbate. For the mentioned reason, carbon dioxide adsorption was also carried out at − 78 °C (see Fig. 3d), which can also be applied to the surface area calculation. Although both molecules are non-polar, they differ in the value of the quadrupole moment 4.7 × 10^–40^ C m^2^ for N_2_ and 14.3 × 10^–40^ C m^2^ for CO_2_^40^. The CO_2_ adsorption isotherm also shows type *I* behavior, similar to the nitrogen as a probe molecule, and the calculated surface area was 198 m^2^ g^−1^. The calculated surface areas are comparable to^54^, which reached values of 203 and 196 m^2^ g^−1^ for N_2_ and CO_2_ adsorption, respectively, and the authors also observed the mentioned limitations. Since the surface values calculated by us from N_2_ and CO_2_ adsorbates are comparable with the published results, we can summarize that we succeeded in preparing STAM-1 with the desired textural properties. It should be noted that the required surface size or pore volume with theoretically calculated values for STAM-1 can be achieved by the adsorption of methanol vapours^54^.

The phase composition and purity of STAM-1 bulk were studied through a powder X-ray diffraction experiment. Figure 3e compares the experimentally measured and calculated PXRD pattern from the crystal structure^54^. The diffraction lines are in good agreement, which indicates the successful preparation of STAM-1. However, parasitic peaks (marked *) can also be observed in the measured PXRD pattern, revealing a small amount of HKUST-1 as an impurity. Using the Rietveld method, the phase composition of the mixture was determined, and the amount of STAM-1 was 98% (2% HKUST-1).

### SEM and EDX analyses

**Figure 4:**
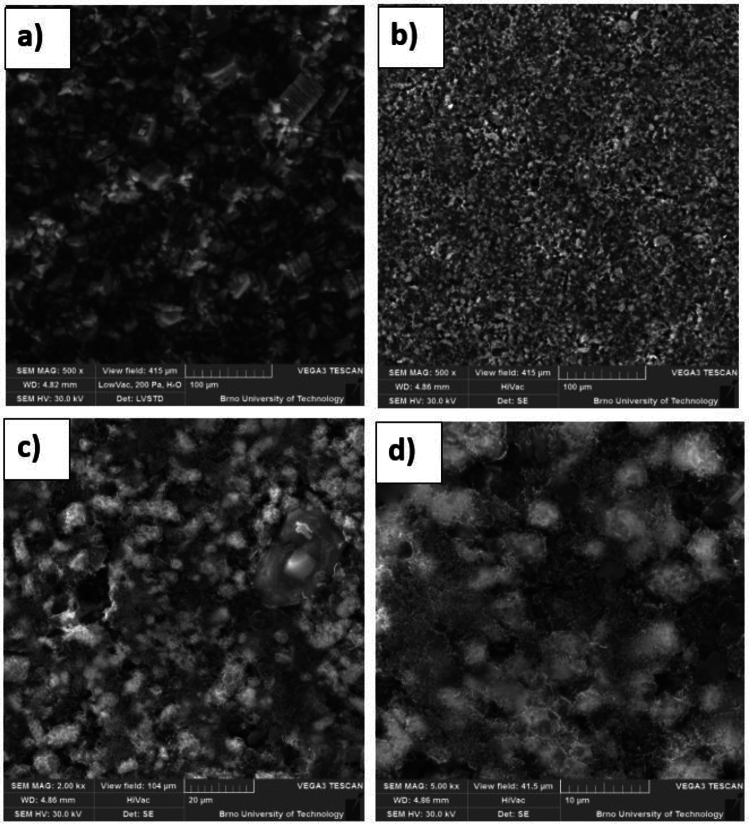
SEM images of the (**a**) STAM-1; S/STAM-1/Super P/PVDF electrode (mill) with magnification (**b**) 500×, (**c**) 2000×, and (**d**) 5000×.

Scanning electron microscopy (SEM) was used to examine the morphology of the prepared electrode's surface. Figure 4a shows an SEM image of the pure STAM-1 with 500 × magnification before use as a precursor for the preparation of the cathode. As can be seen from Fig. 4, STAM-1 creates flake-like structures of different sizes. From about 10 μm for small flakes and about 50 μm for bigger flakes. Figure 4b–d show SEM images of the pressed electrode S/STAM-1/Super P/PVDF prepared by the ball milling method. Images were performed at 500×, 2000×, and 5000× magnification. Figure 4b shows that the surface of the electrode with the material after milling has an even distribution of particles without any cracks. At higher magnification and lower view field (Fig. 4c,d), it is seen that the surface is uniform and porous, while particles that have been formed do not have specific shapes and sizes. However, the size ranges of particles do not exceed 20 μm.

**Figure 5:**
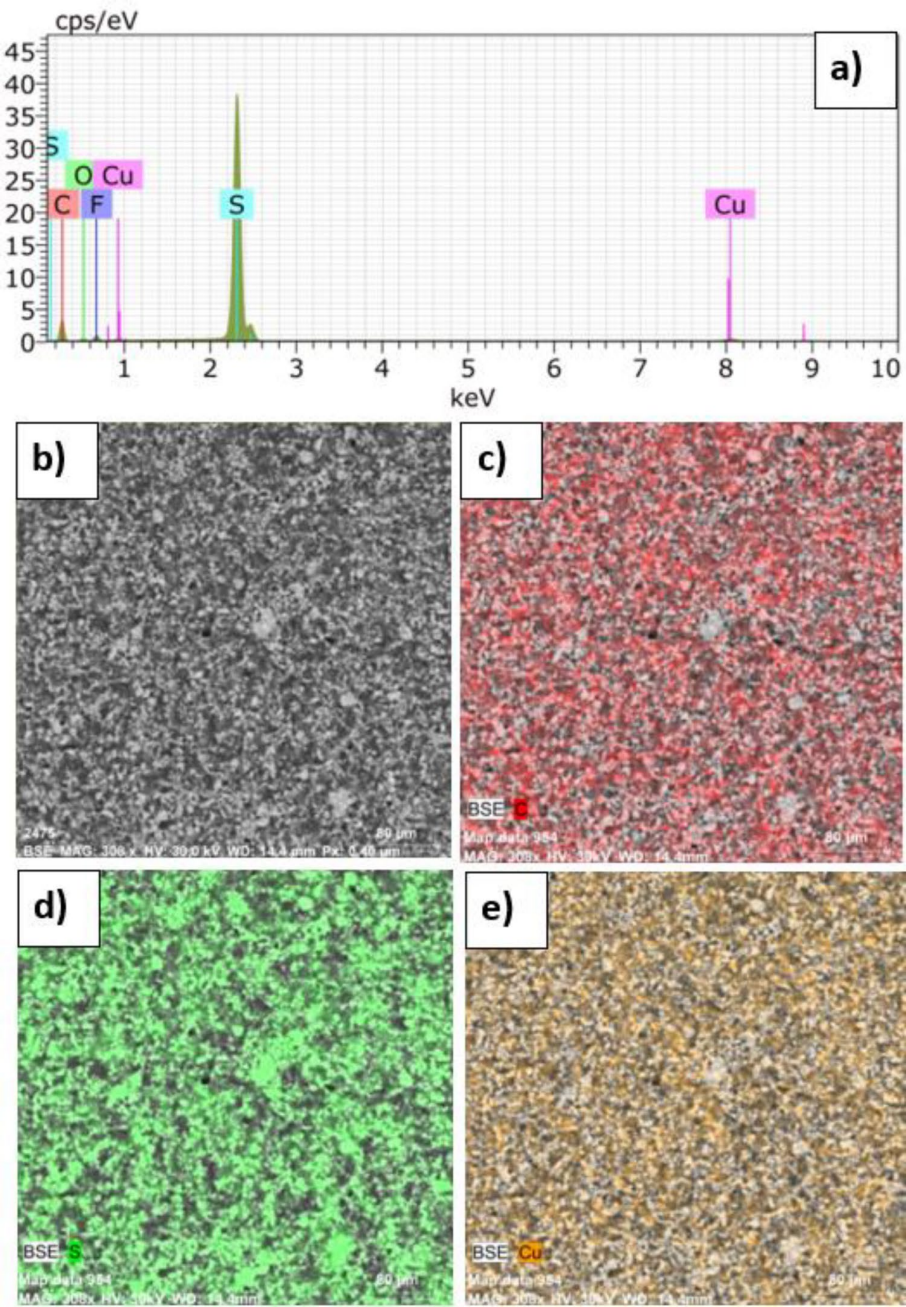
(**a**) EDX spectrum and EDX mapping of the electrode: (**b**) analyzed area, (**c**) distribution of carbon; (**d**) distribution of sulfur, and (**e**) distribution of copper.

For further characterization of the prepared electrodes, EDX analysis was used to determine the composition of the examined sample and the distribution of individual elements in the sample. Figure 5a shows the corresponding EDX spectrum obtained by this analysis. The aluminium is present from the used current collector and fluorine was observed from the used binder PVDF. The presence of copper, oxygen, and carbon is observed due to the STAM-1 and Super P used, which contain mentioned elements in their structures. Figure 5b shows the studied part of the electrode surface by the EDX analysis, and Fig. 5c–e display the homogeneous distribution of carbon, sulfur, and copper.

**Figure 6:**
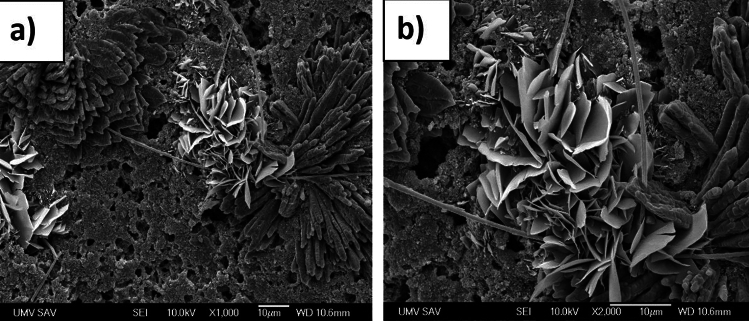
SEM images after cycling with magnification (**a**) 1000× and (**b**) 2000×.

To substantiate the cyclic stability and investigate morphology *of STAM-1* cathode after the cycling process SEM analysis was provided (Fig. 6). SEM images after cycling revealed minor structural changes of *STAM* cathode, which shows, compared to cathode before cycling, more porous surface and slightly altered morphology. However, the overall structure seems to maintain initial composition. EDX analysis confirmed presence of C, O, and F.

Improved capacity retention might be linked to the two types of growths present on SEM images after cycling. First one is composed of thin petals, resembling structure of hematite or malachite. EDX analysis showed presence of carbon, oxygen and sulfur, suggesting creation of oxidated species and/or polysulfides on the cathode surface after cycling. The other structure is composed of more round fiber-shaped crystals, according to EDX mostly carbon and oxygen. This might mean that copper actively reacted during cycling process leaving only C-O skeleton of STAM intact. The electronic kinetics is influenced by presence of carbonyl compounds due to prolonger exposure time increasing probability of electron transfer^60^.

### PXRD analysis

**Figure 7:**
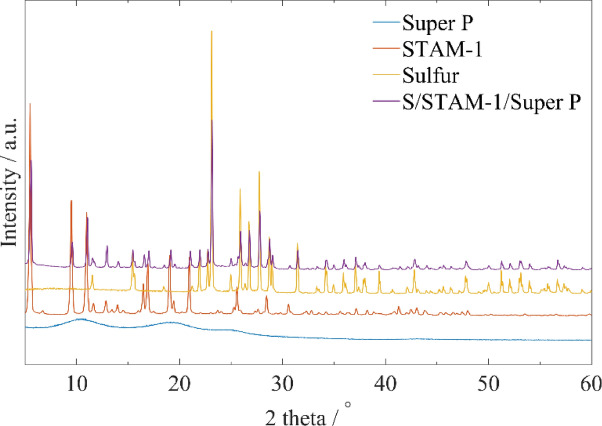
PXRD patterns of carbon super P (blue); STAM-1 (orange); sulfur (yellow) and powder S/STAM-1/Super P (violet).

To investigate the crystal structure, crystallinity, and composition of individual components and prepared electrode, the powder X-ray diffraction (PXRD) analysis was performed, and corresponding PXRD patterns of Super P, STAM-1, sulfur, and S/STAM-1/Super P are shown in Fig. 7. Super P showed broad and diffuse diffraction lines in the PXRD pattern, which reflect the amorphous nature of this material (see Fig. 7 blue line). Measured diffraction lines for pure STAM-1 are consistent with the simulated pattern from the crystal structure, which confirms the successful synthesis and structure of the material (see Fig. 7 red line, Fig. 3e). Also, the measured diffraction record of sulfur is in good agreement with the theoretical pattern for orthorhombic S modification (see Fig. 7 yellow line). The successful preparation of the S/STAM-1/Super P composite is confirmed by the violet line in Fig. 7, as the PXRD pattern contains diffraction lines originating from sulfur as well as STAM-1.

### Raman analysis

**Figure 8:**
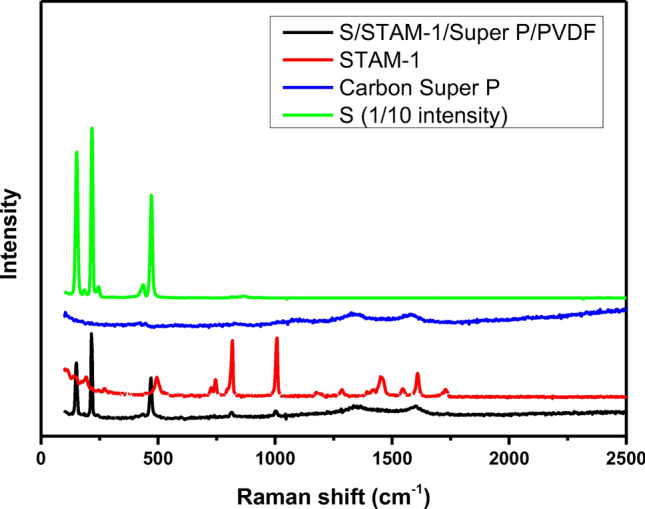
Raman spectra of S/STAM-1/Super P/PVDF (black line), STAM-1 (red line), Carbon Super P (blue line), Sulfur (green line).

Raman analysis was also used for the characterization of STAM-1, carbon super P, sulfur, and S/STAM-1/Super P/PVDF electrode (see Fig. 8). As is seen in Fig. 8 (black spectrum), well-defined Raman peaks were observed. The typical vibration of the sulfur is located at 150, 216, and 470 cm^−1^ (green spectrum), and also satellite peaks with very low intensities at 245 and 434 cm^−1^ are typical for S_8_. Peaks observed at 800 and 1000 cm^−1^ contributed to the C–C stretching mode and breathing mode of the aromatic ring coming from STAM-1, respectively (reference red spectrum). The broad peaks at 1352 cm^−1^ and 1601 cm^−1^ correspond to D and G band vibrations, respectively. The D band is often related to the disarranged carbon structure and deformities such as vacancies, functional groups, etc. and the D band derives from sp^2^ carbon^61^. The peak intensity ratio of the D band/G band (I_D_/I_G_) is 0.94 and 1.08. This intensity ratio describes the degree of arranged carbon^62^.

### XPS analysis

**Figure 9:**
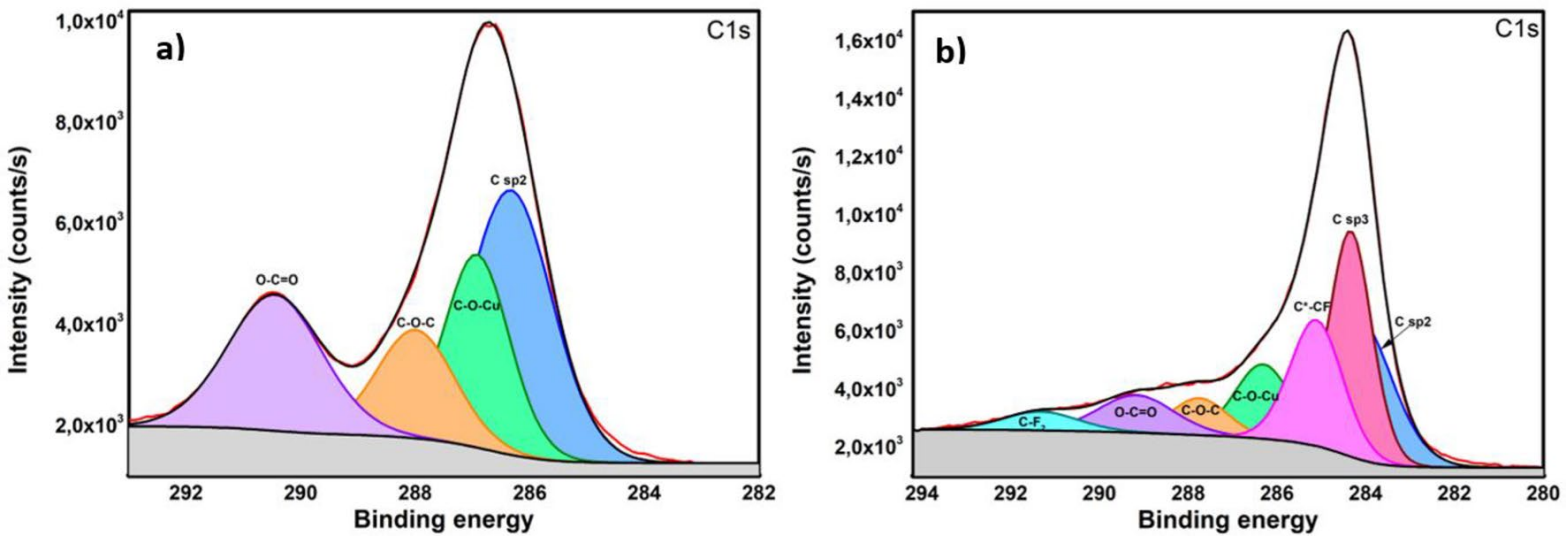
The high-resolution C 1s XPS spectrum of (**a**) STAM-1 and (**b**) S/STAM-1/Super P/PVDF.

For the evaluation of STAM-1 XPS spectra, a comparison with a similar structure (HKUST-1) was performed. Figure 9a shows C 1s XPS spectrum of STAM-1 fitted with four subpeaks corresponding to sp^2^ carbon, C–O–Cu, C–O–C and O–C=O components. Figure 9b shows C 1s XPS spectrum of S/STAM-1/Super P/PVDF composite fitted with seven subpeaks corresponding to sp^2^ and sp^3^ carbon of C*–CF, C–O–Cu, C–O–C, O–C=O and CF_2_ components. The high electronegativity of fluorine and oxygen also affects atoms, which are not directly connected to these elements but are the closest neighbours. Therefore, we assume that the binding energy of the -[H_2_C*-CF_2_]-_n_ carbon atom will be slightly shifted compared to CH_2_ in a different configuration^63,64^. The binding energy of the main C1s peak for STAM-1 is 286.7 eV, and for S/STAM-1/Super P/PVDF composite is 284.4 eV. This shift is related to a significant improvement in the electronic and, thus, the conductivity properties of the composite compared to STAM-1 itself^63,64^.

**Figure 10:**
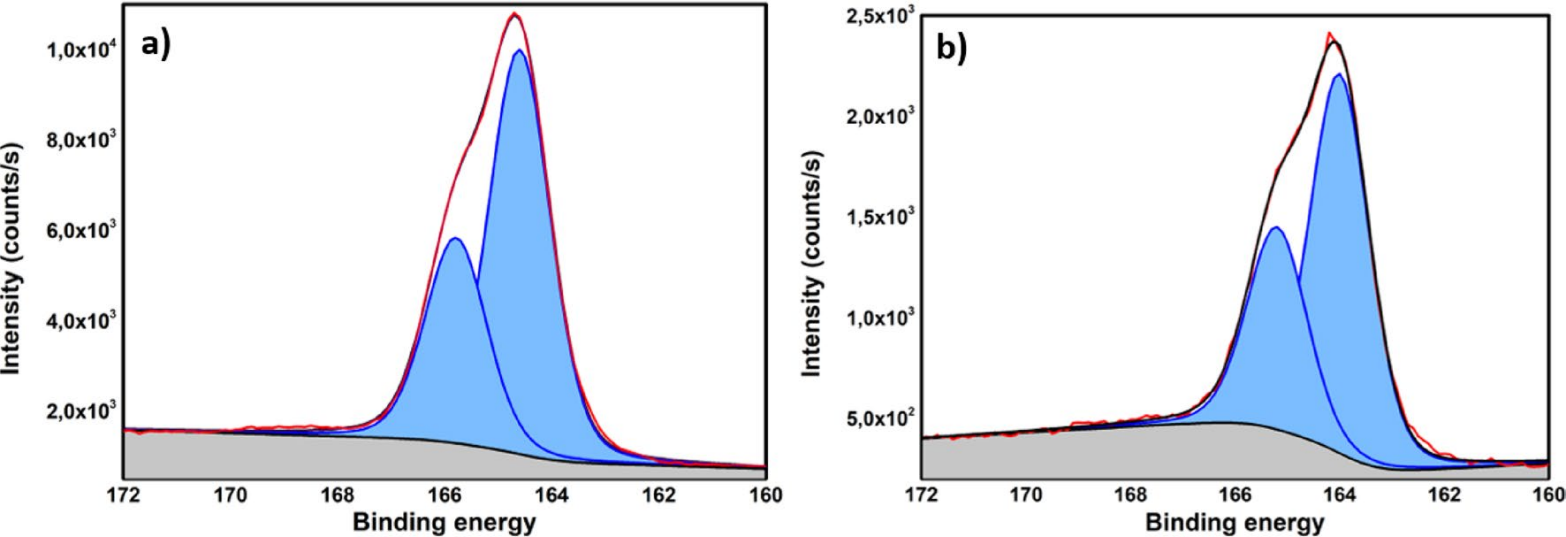
The high-resolution S 2p XPS spectrum of (**a**) octasulfur and (**b**) S/STAM-1/Super P/PVDF.

Figure 10a shows S 2p XPS spectrum of octasulfur (S_8_) fitted with two components corresponding to S 2p3/2 with binding energy at 164.6 eV and S 2p1/2 component at 165.79 eV, with spin–orbit splitting of 1.19 eV.

The parameters obtained from the fit for pure sulfur were used in fitting the S 2p peak of the investigated composite. The S 2p peak of the S/STAM-1/Super P/PVDF composite is shown in Fig. 10b. The main peak was also fitted with components for S 2p3/2 and S 2p1/2 with binding energy at 164 and 165.2 eV, respectively, and with a spin-orbital splitting of 1.2 eV. It could be assumed that the chemical state of sulfur is the same in octasulfur and the S/STAM-1/Super P/PVDF composite. The shift in the position of the sulfur peak for the composite compared to the reference sulfur is related to the better electronic properties of the resulting composite.

### Electrochemical analyses

**Figure 11:**
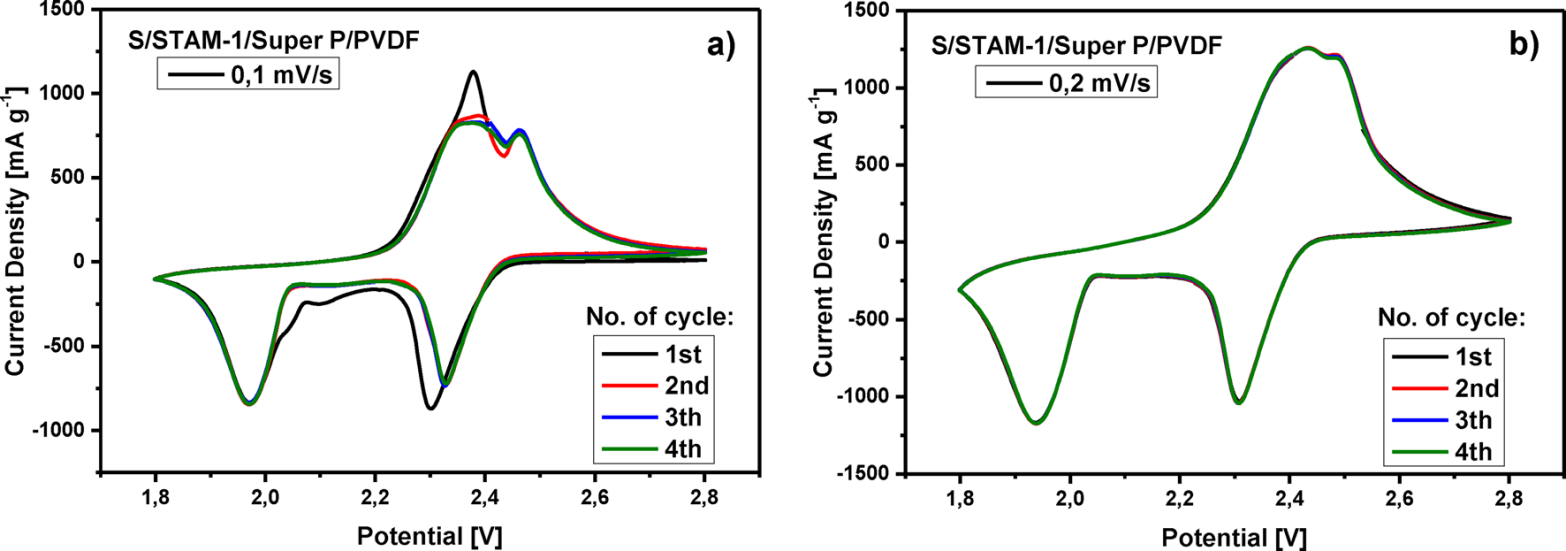
CV curves of S/STAM-1/Super P/PVDF cathode at a scan rate of (**a**) 0.1 mV s^−1^ and (**b**) 0.2 mV s^−1^.

After the structural analyses, the test cells (El-Cell®) were constructed in the argon-filled glove box with a guaranteed oxygen and water content below 10 ppm, and the electrochemical properties of prepared positive electrodes were examined. Cyclic voltammograms of the S/STAM-1/Super P/PVDF electrode were performed in a potential window from 1.8 to 2.8 V and at scan rates of 0.1 mV s^−1^ and 0.2 mV s^−1^ (see Fig. 11). In both cases, two oxidation and two reduction peaks were observed. In the case of cyclic voltammetry curves at a measurement speed of 0.1 mV s^−1^, the oxidation peaks are more significant than oxidation peaks at 0.2 mV s^−1^. Anodic peaks at potentials 2.4 V and 2.5 V correspond to the oxidation processes. Significant two cathodic peaks are observed in both cases at potentials of 2.3 V and 1.9 V, and mentioned peaks correspond to the multistep reduction processes of transformation of elemental sulfur to long-chain polysulfides (Li_2_Sn, 3 ≤ n ≤ 8) polysulfides through S_8_ reduction and lithium sulfide (Li_2_S_2_ or Li_2_S) through short-chain polysulfide reduction. The anodic peaks represent the oxidation reaction of Li_2_S and Li_2_S_2_ to elemental sulfur. Cyclic voltammetry confirmed excellent reversibility of the system that is affected by the application of Cu(II)-based MOF structure with improved lithium ions pathways, better catalytic effect of Cu to lithium polysulfide and enhanced redox kinetics of tested Cu-STAM Li–S cells.

**Figure 12:**
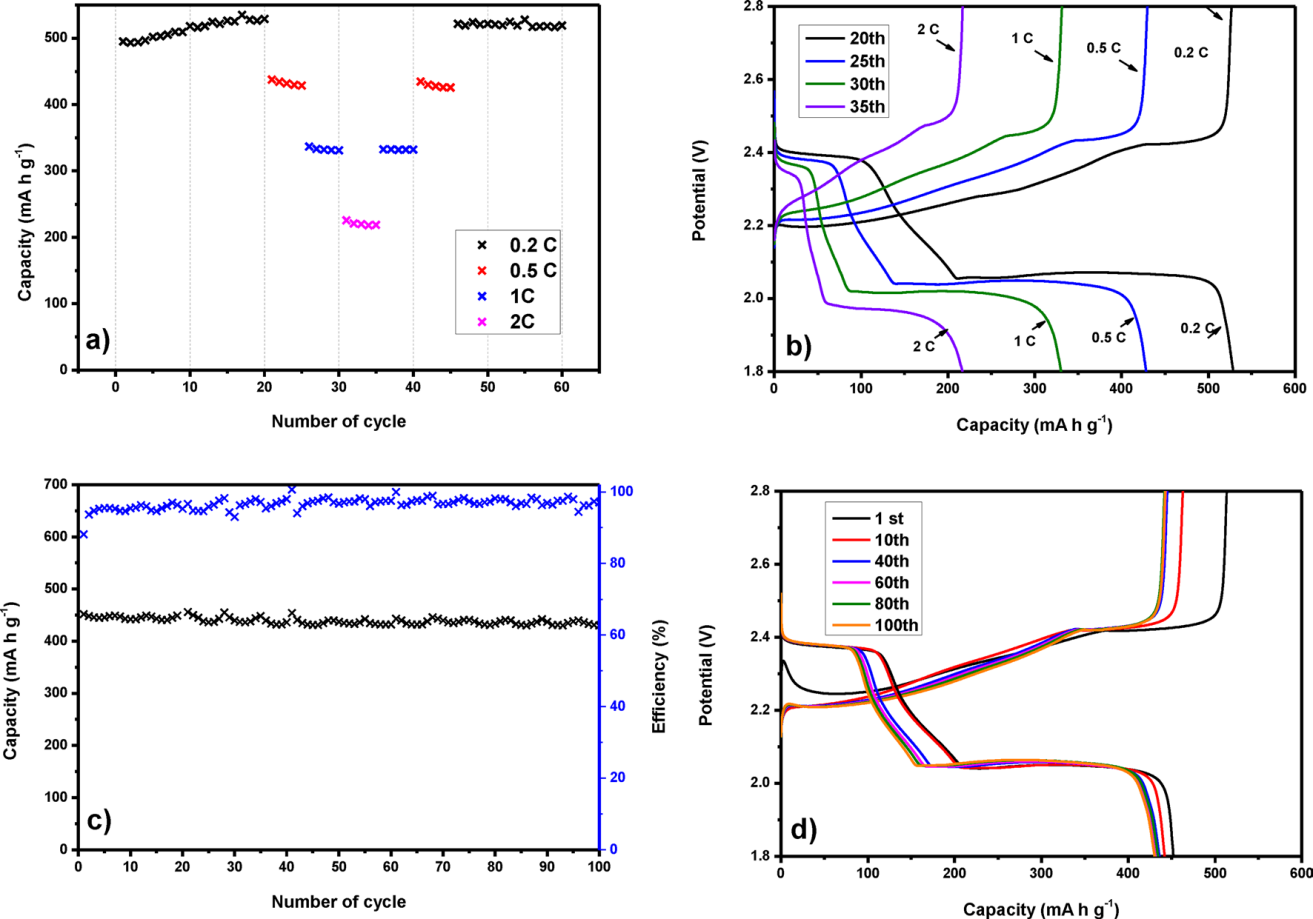
(**a**) Changes of capacity for S/STAM-1/Super P/PVDF at different C-rates. (**b**) Charge/discharge curves of S/STAM-1/Super P/PVDF at different C-rate at the 20th cycle (black); 25th cycle (blue); 30th cycle (green), and 35th (violet). c) Changes of capacity for S/STAM-1/Super P/PVDF at 0.5 C (100 cycles) and d) charge/discharge curves of S/STAM-1/Super P/PVDF at 0.5 C (100 cycles).

The prepared positive electrode was tested by the galvanostatic cycling method at various C-rates. This method is used to determine the behavior of the prepared cathode during the charging and discharging processes. Figure 12a shows the analysis results of capacity changes obtained from galvanostatic cycling, performed at 0.2 C, 0.5 C, 1 C, 2 C, and back gradually stepwise at the same C-rates. With the increase of C-rate, the capacity of S/STAM-1/Super P/PVDF gradually decreases. Electrode shows rate performances with capacities of 529 mAh g^−1^ after 20 cycles at 0.2 C, 428 mAh g^−1^ after another 5 cycles at 0.5 C, 331 mAh g^−1^ after further 5 cycles at 1 C, and 218 mA h g^−1^ after additional 5 cycles at 2 C. Capacities dropping despite multi-current cycling are summarized in Table 1. It is visible from Fig. 12a that during cycling at current densities of 1 C and 2 C, there was no distinct capacity decrease in all cycling processes. Capacity increased after the first 20 cycles at 0.2 C of 6.8% since 100% of the sulfur stored in the porous matrix had not yet been used during the first cycles. However, in the next cycles at 0.5 C, 1 C, and 2 C, the capacity dropped by 13.41%, 33.12%, and 55.84%.

**Table 1:** Capacity evolution during galvanostatic cycling of S/STAM-1/Super P/PVDF at different C-rates.

C-rate	Cycle number	Capacity (mAh g^−1^)	Capacity decrease versus 1st cycle [%]
0.2 C	1	495.2	
0.2 C	20	529	+ 6.82
0.5 C	25	428.8	+ 13.41
1 C	30	331.2	− 33.12
2 C	35	218.7	− 55.84
1 C	40	332.4	− 32.88
0.5 C	45	425.8	− 14.01
0.2 C	50	521.7	+ 5.35
0.2 C	60	519.1	+ 4.81

It is obvious from Table 1 that discharge capacity returns after 60 cycles roughly to capacity at the beginning. It is clearly seen that the capacity after 60 cycles increases versus the first cycle of 4.81%. Figure 12b corresponds to the results of galvanostatic cycling at different C-rates (0.2 C; 0.5 C; 1 C and 2 C) and a different number of cycles. The capacity of the battery decreases with the increasing number of cycles and from 0.2 to 2 C. Discharge curves show two typical plateaus that agree with the mechanism of reduction of sulfur. The high voltage plateau at a potential of 2.4 V corresponds to the conversion of sulfur S_8_ to higher polysulfides, and the low voltage plateau at a potential of 2.0 V corresponds to the reduction of higher polysulfides to lower polysulfides. Figure 12c shows the results of long-term measurements at 0.5 C for 100 cycles with corresponding capacities and Coulombic efficiencies. The initial discharge capacity was 452 mAh g^−1^, and after 100 cycles, the discharge capacity decreased to 430 mAh g^−1^. Coulombic efficiency was around 96% during the whole cycling procedure. Figure 12d shows charge/discharge curves from 1st up to the 100^th^ cycle, where discharge capacity loss is very low and cycling is stable.

**Figure 13:**
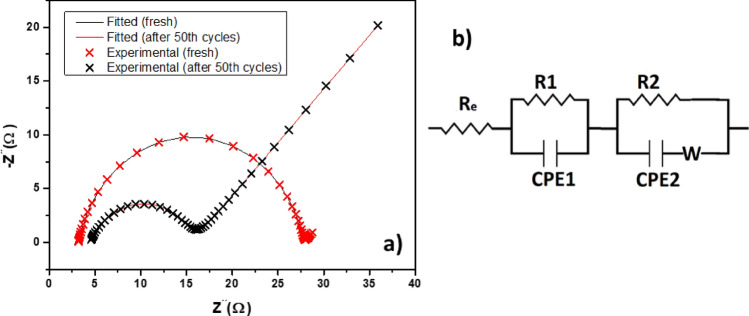
a) Nyquist plot of the S/STAM-1/Super P/PVDF electrode before and after 50 cycles with b) equivalent circuit.

Electrochemical impedance spectroscopy was used to study the electrochemical behavior between the electrode and the electrolyte. Figure 13a,b shows a typical Nyquist plot of the prepared cathode before cycling and after 50 cycles and the equivalent circuit used for the simulation of the EIS spectra. Experimental data are presented as a cross, and simulated data from fitting are presented as a line. The agreement between the measurements and calculated data is high, the error of the simulation fitting before cycling is only 0.04%, and after 50 cycles is 0.16%. Individual elements of the equivalent circuit of the prepared cathode have the following meanings. *R*_*e*_ represents the electrolyte resistance, *R1* is the resistance of the surface layers of the sulfur and lithium electrode, *CPE1* is the capacitance of the surface layers, *R2* is the charge transfer resistance, *CPE2* is the double layer capacitance, and *W* is the Warburg impedance^65,66^. Before cycling, the prepared S/STAM-1/Super P/PVDF cathode shows higher charge-transfer resistance (*R*_*ct*_ = 26.27 Ω) than after 50 cycles at 0.5 C (*R*_*ct*_ = 11.03 Ω). The decrease in the charge transfer resistance after cycling may be due to improved contact of sulfur and matrix, the enhanced position of active material, and better electrolyte penetration into the cathode material. Furthermore, the decrease in Warburg impedance is significant because of the improvement of cathode material wettability using STAM-1 MOF with bimodal pore size and unique shape of skeleton. The obtained results are in agreement with previously published results^67,68^.

The electrode exchange current density (*i*_0_) was calculated with Eq. (1):

1 $${i}_{0}=\frac{RT}{nF{R}_{CT}}$$

In Eq. (1) *R* is the molar gas constant, *T* is the absolute temperature, *n* is the number of electrons transferred in the reaction, *F* is the Faraday constant, and *R*_*CT*_ is the charge transfer resistance. Electrode exchange current density (*i*_*0*_) of the electrode S/STAM-1/Super P/PVDF before cycling is 0.1924 mA cm^−2^, which is lower than after 50 cycles where *i*_*0*_ takes on the value of 0.458 mA cm^−2^.

It is also possible to calculate diffusion coefficients of lithium ion according to the following Eq. (2):

2 $$D=\frac{{R}^{2}{T}^{2}}{2{A}^{2}{n}^{4}{F}^{4}{C}^{2}{\sigma }^{2}}$$

where *A *is surface area, *C* is concentration, and *σ* is Warburg coefficient. Other elements have the same meaning as in Eq. (1)^53^. The diffusion coefficient of a fresh electrode is 9.5 × 10^–10^ cm^2^ s^−1^, and after 50 cycles acquired value is 2.8 × 10^–9^ cm^2^ s^−1^. It is obvious that the diffusion coefficient of lithium ion after cycling increases indicating that STAM-1 cripples sulfur and polysulphides species which arise during cycling, and the shuttle effect can be weakened. Parameters used in both equations are summarized in Table 2. Table 3 presents a comparison between the S/STAM-1/Super P/PVDF and other MOF composite materials used in Li–S batteries.

**Table 2:** Parameters of the S/STAM-1/Super P/PVDF electrode obtained from Eqs. (1) and (2).

	R_CT_ (Ω)	*i*_*0*_ (mA cm^−2^)	D (cm^2^ s^−1^)
Fresh	26.27	0.1924	9.5 × 10^–10^
After 50 cycles	11.03	0.458	2.8 × 10^–9^

**Table 3:** Comparison of S/STAM-1/Super P/PVDF electrode with other MOF composite material used as cathode material for Li–S battery.

Title	S (wt%)	Capacity (mAh g^−1^)		Number of cycles	C-rate	References
		After cycling	Capacity decrease versus 1st cycle [%]			
S@ZIF-8	NA	~ 510	57.5	100	0.1 C	^70^
HPCN-S	NA	730	33.03	50	0.5 C	^71^
S@MOF-525(Cu)	70	~ 700	29.3	200	0.5 C	^72^
HKUST-1 ⊃ S	40	~ 500	66.6	170	0.1 C	^69^
S/MIL/C	60	476	32.5	200	0.5 C	^53^
S/STAM-1/SuperP/PVDF	60	430	4.9	100	0.5 C	This work

## Conclusion

In this work, we have successfully synthesized and characterized the structure and electrochemical performance of cathodic material containing metal–organic framework STAM-1 as a host material for the composite sulfur cathode. The structure of STAM-1 consists of one monomethyl ester of BTC linker (MeBTC(-II)), one Cu(II) central ion, and one coordinated and two crystallization water molecules. Ester functional groups form a hexagonal hydrophobic cavity, and hydrophilic pores are created by the crystallization water molecules. Its unique structure with hexagonal hydrophobic and trigonal hydrophilic pores was effectively absorbed into the electrode material, thus stabilizing and improving the properties of the electrode material, such as volumetric expansion during cycling or polysulphides trapping, which prevented polysulphide shuttle effect. STAM-1 successfully encapsulated sulfur in these pores and thus boosted the electrochemical activity of the cathode. Moreover, the catalytic effect of copper and improved redox kinetics of sulfur cathodes were confirmed. The initial discharge capacity of this prepared cathode (S/STAM-1/Super P/PVDF) was 495 mA h g^−1^ at the current rate of 0.2 C. The initial discharge capacity at the current rate of 0.5 C was 452 mA h g^−1^, and after one hundred cycles, it was 430 mA h g^−1^, with Coulombic efficiency of 97% during the whole cycles at 0.5 C. Finally, these results show that STAM-1 is a promising host material for the sulfur cathode in the Li–S battery. In addition, the combination of the bimodal pore size structure of MOF and Lewis acid metal site is the best combination to create novel MOF-based cathode material with improved stability and performance.

## Data Availability

The datasets generated and/or analysed during the current study are not publicly available due to possible patent application on novel MOF structures but are available from the corresponding author on reasonable request.
